# The human factor in explainable artificial intelligence: clinician variability in trust, reliance, and performance

**DOI:** 10.1038/s41746-025-02023-0

**Published:** 2025-11-14

**Authors:** Angus Nicolson, Elizabeth Bradburn, Yarin Gal, Aris T. Papageorghiou, J. Alison Noble

**Affiliations:** 1 Institute of Biomedical Engineering, Department of Engineering Science, University of Oxford, Oxford, UK; 2 OATML, Department of Computer Science, University of Oxford, Oxford, UK; 3 Nuffield Department of Women’s and Reproductive Health, University of Oxford, Oxford, UK; 4 Oxford Maternal & Perinatal Health Institute, Green Templeton College, University of Oxford, Oxford, UK

**Keywords:** Computer science, Medical imaging

## Abstract

Explainable Artificial Intelligence (XAI) is proposed as essential for high-risk applications like healthcare, where it aims to enhance user trust. However, studies often rely on automated metrics rather than user evaluation. We adapt a prototype-based XAI model for image-based gestational age (GA) estimation and evaluate its impact on trust, reliance, and performance, including a novel measure of appropriate reliance. Ten sonographers completed a 3-stage reader study assessing the XAI model’s impact on GA estimates. Model predictions reduced clinician mean absolute error (MAE) from 23.5 to 15.7 days, and explanations had a further non-significant reduction to 14.3 days. However, the impact of explanations varied across participants, with some performing worse with explanations than without. Additionally, although explanations increased participant confidence, they had no significant effect on trust or reliance on the model. These counterintuitive results highlight potential pitfalls in deploying XAI, emphasising the need for human studies to capture clinician variability.

## Introduction

Deep learning models have been shown to be powerful tools within healthcare, and in imaging are able to achieve performances similar to or surpassing domain experts^1–4^. These models can improve clinician performance when used as advice in clinical decision-making^3,5^. However, in many instances we have little or no ability to understand how models reach their decision—so called “black-boxes”—and this may hamper trust in model predictions^6,7^. To overcome this, Explainable Artificial Intelligence (XAI) has been proposed: here, explanations are provided alongside model predictions, so that trust by end users is enhanced and more detail is given to aid clinical decision-making^8–14^. While explanations can also be used to facilitate debugging during model development^15–17^, the use relevant to this paper is for clinicians to better understand how model predictions or decisions are reached.

There have been many assertions that XAI is required in high-risk scenarios, with an increasing number of researchers calling for XAI in healthcare^18–22^. However, the purported advantages deserve to be examined more closely. Recent studies have called into question the necessity of XAI in healthcare, advocating “rigorous internal and external validation of AI models as a more direct means of achieving the goals often associated with explainability”^23^. Other work has reported the ineffectiveness of model explanations at finding spurious correlations^24^, and how many saliency methods provide explanations that do not depend on their underlying model^25^ or are inferior compared to specialised networks at locating medical abnormalities^26^.

There have been multiple efforts evaluating XAI models through the measurement of some component of interpretability, such as faithfulness (how well the explanations match the causal behaviour of the model); sparsity (how simple the explanations are), and simulatability (how well users can predict a model prediction from its explanations)^8,27–30^. However, the gold standard of evaluating interpretability methods is measurement of performance using real human operators and real tasks^28^.

In this regard, there are currently fewer studies^27,31^. Yu et al.^32^ demonstrate the heterogeneity of the effect of AI-tools on the decision-making of clinicians and that there are no clear predictors of which clinicians will respond favourably (such as years of experience), but they do not examine the effect of model explanations, only model predictions. Gaube et al.^33^ show that task experts did not show a significant improvement in reviewing X-rays, but non-task experts can benefit from model explanations. Other work has shown that explanations can sometimes have a negative effect, with explanations for incorrect model predictions causing clinician treatment decisions to get worse for antidepressant selection^34^. The effect of explanations on human behaviour in clinical decision-making can be difficult to predict with Nagendran et al.^35^ showing no correlation between self-reported usefulness of XAI and influence of explanations on prescription decision-making. Overall, the role of explanations in improving clinical decision-making remains poorly understood. Building on prior work, we examine how sonographers respond to both model predictions and explanations generated by a prototype-based XAI method that outputs explanations in the form of images and heatmaps.

To determine if model explanations are beneficial, we must first clearly define a specific use case and the purpose of the explanations to test if they achieve their stated purpose. In this study we hypothesise that the use of XAI improves user trust (through the provision of explanations), leading to increased reliance on model estimates and improved user performance (since the explanations enhance available information, informing decision-making).

In the analysis of our results, we make a distinction between two related ideas: trust and reliance. We define trust as “the attitude that an agent will help achieve an individual’s goals in a situation characterised by uncertainty and vulnerability”^36^. In the context of this study, the agent is a machine learning model and the individual is a human participant. Whereas reliance is “the extent to which an agent influences an individual”. We can measure the extent of this influence using the change in a participant’s estimate. Trust is an attitude, whereas reliance is a behaviour. Trust guides, but does not completely determine, reliance.

Blind reliance on an inaccurate model can lead to negative outcomes. Instead, we want to achieve *appropriate reliance*, where participants rely on the model when it is correct but ignore it when incorrect^36–39^. In this work, we propose a novel, behaviour-based definition of appropriate reliance that depends on the relative performance of the user and the model. Each estimate can be categorised as:

- Appropriate reliance: participant relied on the model when it was better, or did not when it was worse
- Under-reliance: participant did not rely on the model when it was better
- Over-reliance: participant relied on the model when it was worse

Importantly, this definition of appropriate reliance does not assess whether a participant’s estimate improved, but rather whether their behaviour was justified given the model’s relative performance.

As our use-case, we examine gestational age (GA) estimation from fetus ultrasound. Many of the proposed clinical imaging applications of AI are in radiology so this provides a useful example, especially as it has recently been shown that AI (deep-learning image-based automated GA estimation) can be more accurate than current clinical practice (manual biometry)^40^.

It has been noted that “there does not appear to be a consensus regarding a validation protocol, which hinders the progress of explainable ML research by making explainable methods incomparable”^8^. We aim for our study design to become a valuable tool for evaluating XAI methods for healthcare applications. Using a three-stage design, we measure:

- The clinician’s decision-making process without AI
- The influence of model predictions
- The additional influence of model explanations

Currently, the most common form of explanation used in XAI for medical imaging is saliency^41,42^, which visualises the gradient of the model output with respect to the input pixels. This gives a heatmap of which regions of the image the model is most sensitive to. However, saliency maps lack important detail, only showing ‘where’ the model focused in the image, and not ‘what’ it focused on^25,43^. Other types of XAI models use different ‘cognitive chunks’^44^, i.e., rather than using pixels to explain a model, they use something else. For example, case-based reasoning^45^ or counterfactual methods^46^ explain models using whole images, or concept-based methods explain a model’s response with respect to semantically meaningful concepts^47–49^. However, counterfactual methods rely on generative models that offer limited guarantees for producing plausible and clinically meaningful counterfactuals^50,51^, while concept-based methods typically require pre-defined, manually annotated concepts^47^. Currently, there is no consensus in the literature on what form of explanation is most suitable for clinical settings^41,52^, but in this work, we utilise a part-prototype model^45,53^ which classifies an image by comparing it to sub-parts of images it saw during training. This provides an explanation similar to how a clinician might make a prediction, e.g., “this foetus is 30 weeks of gestation, because it looks like a 30-week foetus I have seen before”. Using this type of XAI model allows us to utilise the algorithmic complexity and performance benefits of deep learning, not requiring any pre-defined concept labels, while providing more detailed explanations than a simple saliency map.

## Results

A total of 10 clinicians (nine sonographers and one obstetric registrar) participated in the study (Table 1). Each participant evaluated 65 images in each stage and all participants completed all three stages of the study.

**Table 1:** Participant demographic information, collected prior to stage 1

Age / years	<25	25–34	35–44	45–55	>55
	0	4	3	1	2
**Job Title**	Sonographer	Obstetric registrar			
	9	1			
**Region of the UK**	East of England	London and the South East	Midlands	North West England	
	1	7	1	1	
**Experience in foetal ultrasound/years**	< 2	2–5	6–10	≥10	
	1	2	3	4	
**Obstetric scan frequency**	Daily	Weekly	Fortnightly	Monthly	Infrequently
	7	2	1	0	0

### Performance impact of XAI

Performance at GA estimation improved when participants had access to model predictions with a significant decrease in the mean absolute error (MAE) between stages 1 and 2 (23.5 days, SD 4.3 days vs 15.7 days, SD 6.6 days, *p* = 0.008 by independent T-test, Cohen’s *d* = 1.47) but there was no significant change when participants were given access to model explanations in stage 3 (15.7 days, SD 6.6 days vs 14.3 days, SD 4.2 days, *p* = 0.6 by independent T-test, Cohen’s *d* = 0.26).

### Variability in responses to XAI

**Fig. 1:**
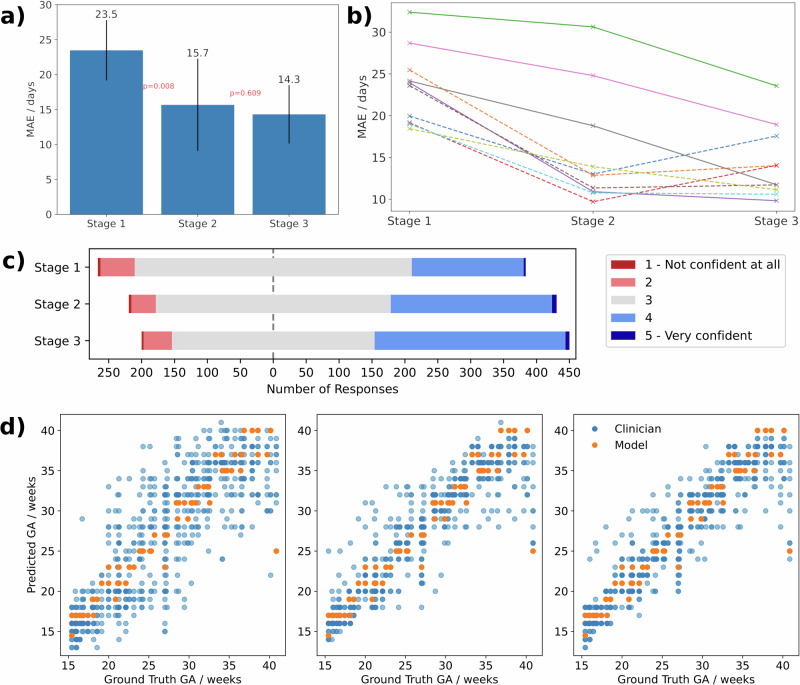
Participant performance and confidence across each stage. The mean absolute error (MAE) at each stage for each participant are shown in aggregate **a** (p values are for adjacent stages, error bars show SD) and for individual participants **b** (solid and dashed lines are participants who self-reported that the explanations were/were not helpful, respectively). The centre **c** shows self-reported confidence for GA estimates on a Likert scale over the three Stages. The bottom **d** show estimated GA for participants (blue) and the model (orange) against the ground truth for Stage 1 (left), Stage 2 (middle) and Stage 3 (right).

**Fig. 2:**
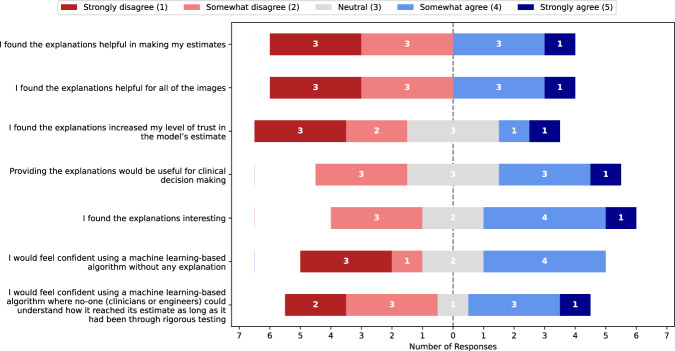
Bimodal opinions on how useful explanations are. Responses to “on a scale of 1–5, how much do you agree with the following statements?” immediately after Stage 3.

However, the mean response of participants hides some detail, with individual participants responding differently to the model explanations. Agreement with model predictions substantially differed between participants (Fig. 5), with some participants performing worse in Stage 3 (Fig. 1, top right panel) while others improved. In exploring factors which could account for this difference in behaviour we found a significant negative linear association between the participants agreement to the following statement: “I found the explanations helpful in making my estimates” after stage 3 (Fig. 2) and their change in MAE between stages 2 and 3 (*r* = −0.74, *p* = 0.014 by linear least squares regression). A negative association indicates that the participants who found the explanations helpful had a reduction in MAE between stages 2 and 3, i.e., an improvement in their performance. This question was not asked in isolation, and a bimodal response can be seen to similar questions about the usefulness of the explanations in stage 3 (Fig. 2).

In order to understand why some clinicians performed worse, the association between participant characteristics (age, clinical experience, opinions/experience with AI prior and post study) and changes in MAE between stages 2 and 3 was evaluated. The only significant features (by linear least squares regression) were responses to questions in the post-stage 3 questionnaire related to the participants’ opinions of the explanations (the top three questions in Fig. 2). Similarly, when comparing participants who found explanations useful versus those who did not, the only factors for which there was a significant difference (*p* < 0.05 by independent T-test) were responses to questions about the explanations themselves. This indicates that no information available prior to completion of stage 3 makes a good predictor for whether a participant will benefit from explanations or not. For a full list of the factors analysed and complete statistical results see Supplementary Note 3.

Figure 5 shows that participants agreed with the model 70% (29% SD) of the time with access to just the predictions and 73 (22% SD) of the time with model predictions and model explanations. This is not a significant difference (*p* = 0.77 by independent T-test) but, once again, solely observing the mean obscures important detail. Figure 5 shows there were substantial differences between participants. For example, participant 1 had low agreement that did not substantially change (29–37%), participant 0 had low agreement that increased substantially between stages (49–98%), participants 4 and 6 had perfect agreement in both stages, and participant 8 had high agreement which dropped in stage 3 (97–69%). The variation in both the initial agreement in stage 2 and the change in agreement in stage 3 highlight the varied responses that clinicians can have upon receiving XAI advice.

A wide variety of image features were used by participants to estimate GA (Supplementary Fig. 2), with a mean of 2.76 (1.26 SD) features per image and a mean of 7.8 (1.32 SD) features used by each participant. This demonstrates that not only did the participants have a wide range of responses to model explanations, but they also provided a wide range of explanations for their own estimates.

### Participant confidence

Participants ranked their confidence in their own estimates for each image on a Likert scale of 1–5 (where 1 is “not confident at all” and 5 is “very confident”). Participant confidence increased between stages 1 and 2 (3.18, 0.59 SD vs 3.33, 0.63 SD, *p*«0.001 by Wilcoxon signed-rank test) and between stages 2 and 3 (3.33, 0.63 SD vs 3.39, 0.65 SD, *p* = 0.014 by Wilcoxon signed-rank test). If the data is split by whether participants indicated they found the explanations helpful (Fig. 4), the distribution of confidences in stage 3 are significantly different across the two groups of participants (*p* = 0.021 by Mann-Whitney U test) but there is no significant difference for stage 2 (*p* = 0.099 by Mann-Whitney U test).

### Trust and reliance

The GA estimates of individual participants became closer to model estimates once participants were shown model predictions in stage 2 (Fig. 1, bottom panels and Fig. 5), indicating the participants were relying on the predictions. This is apparent in the substantial change in agreement with the model between stages 1 and 2 (0.35, 0.10 SD vs 0.70, 0.29 SD, p = 0.0022) and the weight of agreement (WoA) of 0.65 (0.27 SD) indicates that participants’ stage 2 estimates are closer to model estimates than their initial estimates in stage 1 (see Table 2 and the Methods section for an explanation of WoA). However, the addition of explanations in stage 3 had no significant effect on mean WoA (0.65, 0.27 SD vs 0.71, 0.20 SD, *p* = 0.58 by independent T-test) or mean agreement (0.70, 0.29 SD vs 0.73, 0.22 SD, *p* = 0.77 by independent T-test).

**Table 2:** An intuitive interpretation of different values of the weight of advice (WoA) metric

WoA Value	Interpretation
<0	The participant’s estimate moved further away from the model’s estimate.
0	The participant did not change their estimate.
0.5	The final estimate is the mean of the initial estimate and model estimate.
1	The final estimate matches the model’s estimate.
>1	The participant’s estimate moved towards the model estimate, but they overshot, and their final estimate was beyond the model’s estimate.

In general, a higher WoA indicates greater reliance, although this assumes that participants rarely overshoot.

**Fig. 3:**
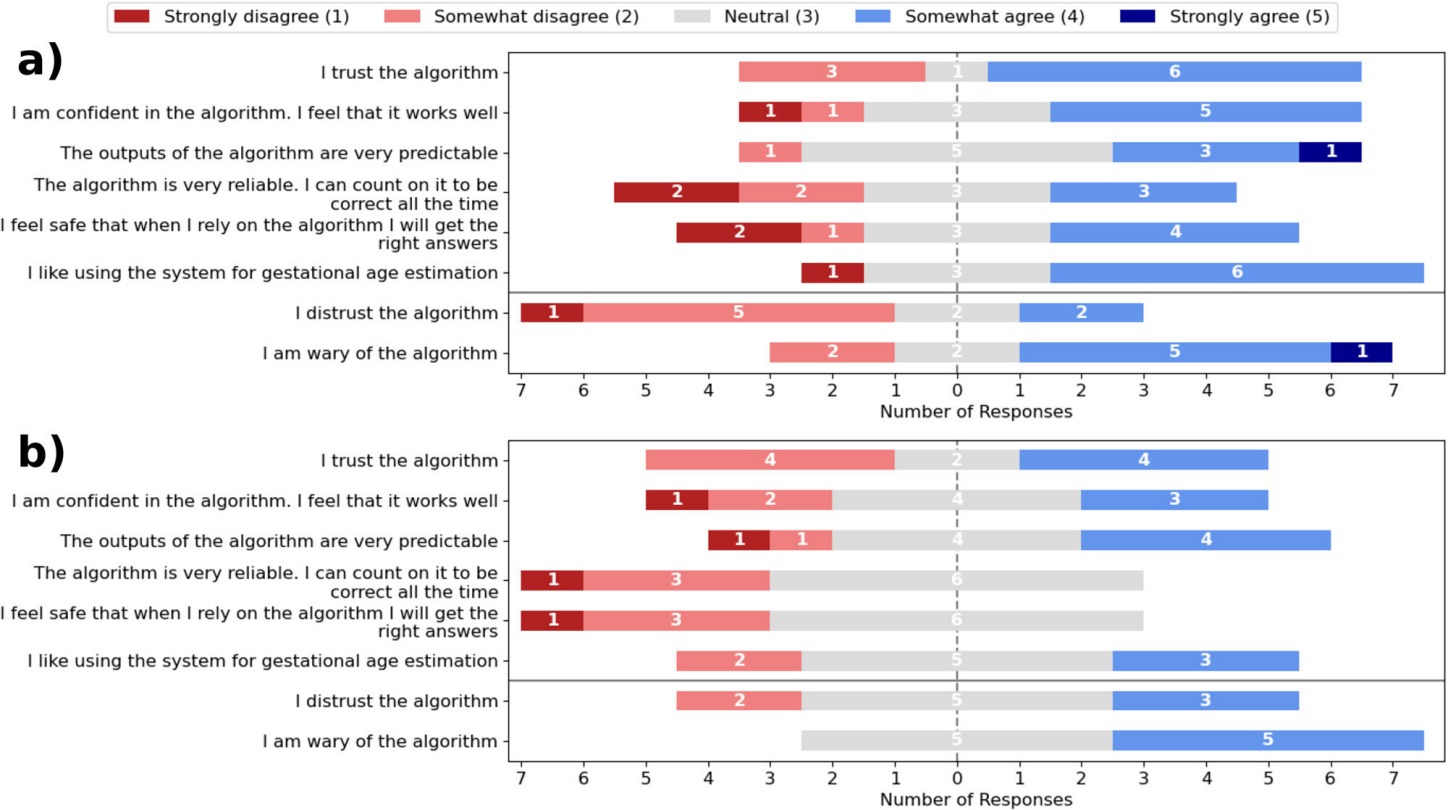
Participant trust in the model with and without explanations. Responses to: “on a scale of 1-5, how much do you agree with the following statements?” for Stage 2 **a** and Stage 3 **b** on a Likert scale.

Similarly, there is no significant change in self-reported trust between stages 2 and 3. After both stage 2 and stage 3 the participants completed a questionnaire ranking their agreement to different statements relating to their trust in the model on a 1–5 Likert scale (Fig. 3). Although the general shift of responses in Fig. 2 is towards decreased trust (a mean change of –0.30 and SD of 0.16 across all questions), there is no significant difference for any individual statement. For example, there was no significant change in agreement to the statement “I trust the algorithm” (3.30, 0.95 SD vs 3.00, 0.94 SD, *p* = 0.45 by Wilcoxon signed-rank test) or “I distrust the algorithm” (2.50, 0.97 SD vs 3.10, 0.74 SD, *p* = 0.084 by Wilcoxon signed-rank test) even with a mean change in response of –0.3 and 0.6, respectively.

There was no significant difference in appropriate reliance between stages 2 and 3 (65.8%, 8.5% SD vs 69.2%, 6.2% SD, *p* = 0.3 by independent T-test). However, within stage 2 there was more than 3-times the amount of appropriate reliance than under-reliance (log ratio of 1.21, 0.72 SD, *p* < 0.001 by dependent T-test) and no significant difference between under-reliance and over-reliance (log ratio of 0.59, 0.98 SD, *p* = 0.092 by dependent T-test). In stage 3, there was more appropriate reliance than under-reliance (log ratio of 1.29, 0.51 SD, *p* < 0.001 by dependent T-test) and more than twice the amount of under-reliance than over-reliance (log ratio of 0.65, 0.68 SD, *p* = 0.014 by dependent T-test).

### Clinicians’ opinions on AI and explanations in clinical practice

Some participants had a less positive opinion on using machine learning in clinical practice after the study (Supplementary Fig. 1). At the beginning and end of the study, we asked whether participants agreed with a variety of statements, including: “I would be comfortable incorporating machine learning based algorithms for GA estimation into my clinical practice”. Participants ranked their responses on a 1–5 Likert scale (with higher numbers indicating increased agreement with the statement), and there was a significant decrease after the study (mean 4.30, 0.67 SD vs 3.50, 0.97 SD, *p* = 0.046 by Wilcoxon signed-rank test).

### Explanations did not slow participants

There was no significant difference in the time participants took to estimate the GA of each image between stages 2 and 3 (17.2 s 13.0 SD vs 19.6 s 10.5 SD *p* = 0.66 by independent T-test). The times for stage 1 cannot be compared, because the participants were asked to complete more information for each scan in stage 1 than in stages 2 and 3.

## Discussion

In this study, we examined the impact of XAI on trust and performance in ultrasound image-based GA estimation. We found that AI advice in the form of the model estimate reduced participant MAE from 23.5 days to 15.7 days and although additional advice in the form of model explanations slightly improved the MAE further the difference was not significant. The improvement in GA estimation with access to model predictions is unsurprising due to the difference in accuracy of the participants and the model in isolation: a MAE of 23.5 days (19.8 days SD) and 9.4 days (14.4 days SD), respectively. An appreciation of the task under consideration is important here. The participants had been asked to perform a task which, although related to clinical care, is not one they have been trained to do, and so they may be more likely to defer to AI advice. In fact, it is surprising they did not defer to AI guidance more often, with a mean agreement of 70 and 73% for stages 2 and 3, respectively.

Solely observing the mean difference in participant MAE and agreement hides important details. The effect of explanations substantially varied among participants, with some participants seeing an improvement and others a decline in performance. Whether a participant improved from explanations or not was correlated with their self-reported opinion on how helpful they found the explanations. This suggests that participants were aware of when explanations helped or hindered them, contrasting with previous literature suggesting that self-reported usefulness of XAI is not a good predictor of clinician behaviour^35,54^.

The individualised response to both the model predictions and explanations highlights the importance of studies such as this. Metrics which evaluate the performance or interpretability of an XAI model cannot shed light on how humans might respond to the advice or the differences between these responses. If XAI models are to be used in clinical practice, it is vital that they are evaluated with the clinicians who would use the tool and the clinicians’ responses carefully examined.

Explanations had no significant effect on participant self-reported trust in the model (Fig. 3). If any signal is present, it points towards a decrease in trust. For example, the median change in agreement to “I distrust the algorithm” was +0.5 and was close to significant (p = 0.084 by Wilcoxon signed-rank test). This is the antithesis of our hypothesis in the introduction and the common assertion that “by enhancing the interpretability of a system, trust from an expert user will also be enhanced”^6,12^. We hypothesise that some participants had reduced perceived trust because of a mismatch between the explanations presented to the participants and their method of estimating GA by eye. This hypothesis is supported by the participants responses to “In what way did the explanations provided in stage 3 influence your decision-making?” in Table 3. Although some participants found the explanations “helped in estimation” and “improved my level of trust and understanding” others “found the explanations very confusing”, “made me lose interest in what the algorithm thought” or noticed differences in how the model makes predictions compared to a clinician: “you cannot analyse that area with the naked eye but maybe the algorithm can”.

**Table 3:** Participant opinions on the model explanations

Participant ID	ΔMAE (Stage 3 – Stage 2)	In what way did the explanations provided in stage 3 influence your decision-making?
0	-7.1	It helped me see the area of the image the model was looking at and this improved my level of trust and understanding.
1	-7.1	The algorithm predicted where I should look make my decision on the GA. However, on occasions, it was completely off
2	-5.9	I would feel more confident scanning in real time and use a TCD as a guide.
3	-2.8	To be honest they didn’t really - I couldn’t understand why some explanations related to gestations completely different from what was being looked at.
4	-1.1	Helped in estimation.
5	-0.2	I found the explanations very confusing, the heat maps and boxes often bore no relationship to the test image so I found it difficult to use ‘this looks like that’. Sometimes the box analysed an occiput and sometimes the frontal bone yet the test image was not in the same position. To assess areas in the anterior/ superior portion of the internal skull where there is so much artefact and reverberation you cannot analyse that area with the naked eye but maybe the algorithm can. It was confusing to analyse.
6	+0.4	I did not find them particularly useful, if the algorithm has been proven to be accurate I feel this is as much explanation as I need to use it confidently.
7	+1.2	The explanations did not make clear sense and therefore [I] relied on my own skill and estimates.
8	+4.4	They made me lose interest in what the algorithm thought so [I] did not reveal its predictions.
9	+4.6	It was interesting seeing visually how the AI concluded GA.

Free text response to how useful the explanations were in stage 3 alongside the participants’ change in mean absolute error (MAE) between stage 2 and 3 (a negative value indicates the MAE decreased in Stage 3, which is an improvement).

**Fig. 4:**
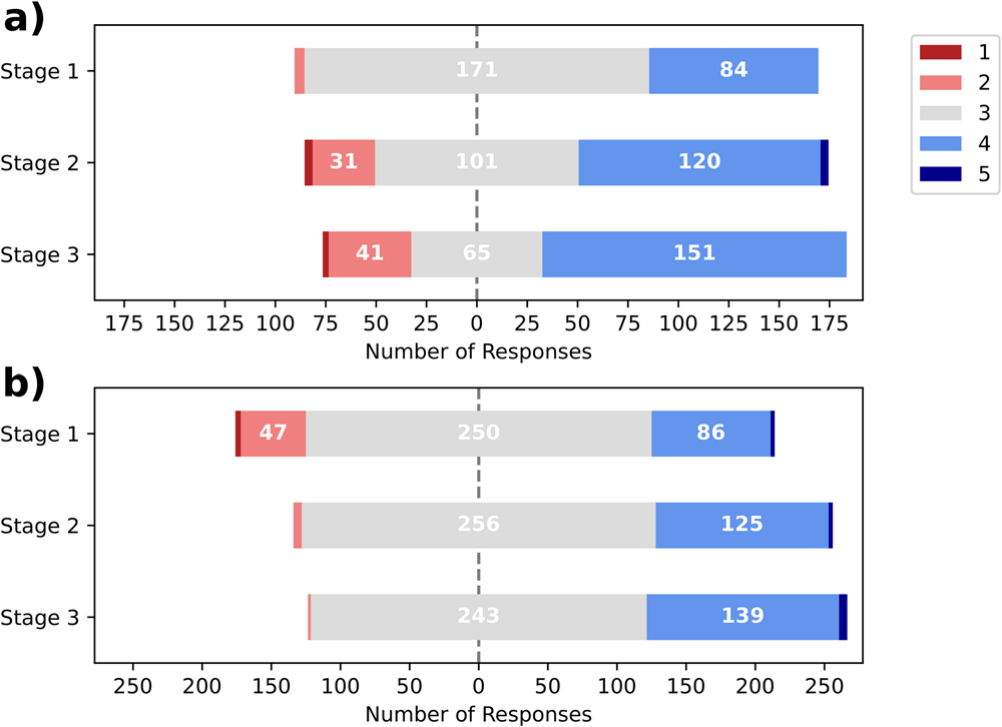
Participant confidence across stages differed based on whether they found the explanations helpful. Participant confidence in their GA estimates - split by if the participant reported to find the explanations helpful (**a**) or not (**b**).

Even though there was no significant change in reliance or self-reported trust in Stage 3, participant confidence increased across stages (Fig. 1). Figure 4 provides some nuance to this story. It indicates that participants who found the explanations helpful had a different response from those who did not. The model predictions caused both groups to increase their confidence between stages 1 and 2. Between stages 2 and 3, however, the participants who found the explanations helpful became both more and less confident with more scores of 2 or 4 as opposed to 3. The remaining participants did not have a large change in confidence and seemed more neutral with most estimates receiving a middle score of 3. This suggests the participants who found the explanations helpful were using them to calibrate their confidence, whereas the others may simply have been more confident in stage 3 because it is the third time they have completed the task.

The study highlights potential benefits and pitfalls of deploying XAI models in clinical settings. The reduction in MAE suggests AI models can enhance clinicians’ estimation accuracy, potentially leading to better patient outcomes. However, the lack of improvement in self-reported trust despite improved performance indicates a need for explanations that are better aligned with clinicians’ expectations and reasoning processes. Moreover, the heterogeneous responses observed across participants point to a broader challenge: how to design explanation strategies that support reliable clinician-AI collaboration across diverse users.

Rather than assuming heterogeneity is irreducible and necessitates personalisation, it may reflect a misalignment between explanation design and clinician reasoning. From this perspective, instead of tailoring explanations to individual users, it may be preferable to identify formats that elicit more uniform responses—those that align better with clinicians’ decision-making processes (e.g., by linking explanations to diagnostic guidelines), reduce interpretive ambiguity via interface design, or are accompanied by training that clarifies their use. Such standardisation could support more consistent responses in practice.

Prior work offers concrete directions: prior information about model accuracy can influence reliance^55^, introducing cognitive forcing functions to encourage participants to examine explanations can reduce overreliance^56^, and adapting assertiveness to experience can reduce errors^57^ and the time taken to diagnose patients^58^. Building on this, future studies should explore what factors related to how explanations are presented to participants (including the form of the explanation, the prior information provided to participants, and the training on their use) can reduce inter-user variance in trust, reliance, and performance. While personalisation remains an important research direction, achieving robust explanation strategies with predictable effects across clinicians is critical for the safe and scalable deployment of XAI in healthcare.

The explanation format used in this study—image heatmaps and prototype comparisons—was unfamiliar to participants; none reported prior experience using heatmaps for image analysis (Supplementary Fig. 1). This unfamiliarity may have made the explanations harder to interpret and increased the mental effort needed to use them. Clinicians are used to reasoning with measurements and anatomical landmarks, while the explanations here relied on visual similarity (“this looks like that”), which may not match their usual way of thinking. This mismatch between explanation format and established mental models can increase extraneous cognitive load (effort spent on understanding the interface rather than the task) ultimately impairing performance or undermining trust. While we cannot confirm this in the present study, future work should incorporate explicit measures of cognitive load to test this hypothesis. Prior work has shown that even in simplified tasks, participants were less able to correct AI model errors when explanations added cognitive burden, despite being more interpretable in principle^59^. In the clinical context, Ghassemi et al.^23^ and Ehrmann et al.^60^ argue that the intended benefits of XAI can be undermined if the explanations increase cognitive load. Relatedly, Asgari et al.^61^ show that high cognitive load from digital tools, such as electronic health records, can contribute to clinician burnout, emphasising the need to design AI systems that reduce rather than add to mental effort. Future work should focus on finding explanation formats—and training or interface designs—that are easier for clinicians to use, rather than assuming more information will be beneficial by default.

Our findings on the diverse impacts of XAI explanations reveal a potential gap in current regulatory frameworks, which often approach AI as a standalone product rather than as part of a complex human-AI system^62,63^. This disconnect is evident in emerging regulations like the European Union’s (EU’s) AI Act^64^, which alludes to the preference of XAI for high-risk AI systems by requiring human oversight to be able to “correctly interpret the high-risk AI system’s output, taking into account, for example, the interpretation tools and methods available”. While this requirement assumes explanations will improve human oversight, our results demonstrate that explanations may actually degrade performance for some users. Regulatory bodies may need to develop new evaluation frameworks that assess not only algorithmic performance but also how effectively explanations support different types of users, potentially incorporating both technical validation of XAI methods and empirical validation of their effects on diverse clinical users^65^.

This study’s main strength is that it underscores the importance of designing rigorous evaluation frameworks for XAI in clinical settings, moving beyond traditional performance metrics to consider human factors like trust and reliance. The study’s design has real-world relevance, in that it closely mirrors a real clinical scenario, providing valuable insights into how XAI might be integrated into everyday clinical practice. The comprehensive analysis is another strength, with a three-stage reader study that allowed for detailed examination of performance, trust and reliance, offering a nuanced understanding of XAI’s impact.

One weakness of the study is that it was conducted with ten sonographers. While this was sufficient to see the differences in approach and value of XAI among this group of clinicians, the small sample size limits generalisability. First, participants were not experts in the specific task, and as reflected in Stage 1 (Fig. 1), their baseline estimates varied substantially. Second, sonographers may differ in their responses compared to other specialties, such as radiologists or emergency physicians, or when analysing different imaging modalities. Finally, the study captures only immediate reactions to XAI. In a realistic clinical scenario, a clinician might be using an XAI tool for many months or years. Longer-term studies will be beneficial to understand how trust and performance evolve over time with continued use. These should include repeated exposures, potentially with training interventions, to assess whether clinicians become more adept at using explanations or recalibrate trust over time.

Our study reveals that while XAI has the potential to enhance performance in GA estimation, its impact on human trust and reliance for this task is complex and variable. In the context of GA estimation, explanations both improved and hindered performance, depending on the clinician. This variability underscores the need for human studies, as it can only be identified through direct observation of how users interact with these systems.

Future research should conduct larger studies across a range of explanation types, clinician specialties, and medical tasks. In particular, comparisons across specialities like radiology or emergency medicine may help identify contexts where different explanation formats are more or less effective. Not only will future studies help determine the generalisability of our findings, but they will also allow researchers to explore why some clinicians benefit from explanations while others do not. Understanding these differences is crucial for refining XAI tools so that they are both trustworthy and effective in clinical practice. In particular, future work should investigate why explanations caused some participants to trust the model less and perform worse, while also developing explanations which better match the internal reasoning of clinicians.

AI is increasingly being integrated into healthcare, and researchers need to understand how different users interact with these systems. Optimising training, user interfaces, and other human factors alongside the development of the explanations themselves will be essential for ensuring that XAI methods support clinicians in making better decisions. This study provides a framework for evaluating XAI in real clinical tasks, highlighting the interplay between trust, reliance, and performance.

## Methods

### The AGE study

We designed the Algorithmic Gestational age Estimation (AGE) study to explore the decision-making process of clinicians when estimating GA from images without biometry and to understand how their behaviour changes with access to an XAI model. At the outset clinicians completed a questionnaire related to their clinical experience, opinions on AI, and demographic information. The study consisted of three stages, where at each stage a participant is asked to estimate GA from an ultrasound image of the foetal head but with successively more information.

In stage 1, participants were asked to estimate GA from an ultrasound image and to rank their confidence on a Likert scale. The participants were also asked to highlight the regions they found most useful and to select the relevant features from a list of options (see Supplementary Fig. 2) or via a free-text option.

After at least 24 h participants were asked to complete stage 2, in a similar manner to stage 1, but a GA estimate from the model is provided alongside the ultrasound image. We asked again for a GA estimate and confidence, but participants were not required to label important features. We also asked questions on trust and how participants used the model estimates.

After a further 24 h or more, stage 3 was undertaken in the same manner as stage 2, but with model explanations in addition to the predictions—see Supplementary Fig. 3 for an example. Again, questions related to their level of trust in the model and how they used the predictions/explanations were asked.

Participants received written instructions and demonstration videos for each stage, including information on how to interpret the model explanations in stage 3. We avoided dictating how participants should use the explanations to inform their estimates, to ensure we did not unduly influence how the clinicians interacted with the system. Instead, we used the phrase “this looks like that” to explain how to interpret the explanations, i.e., regions highlighted in the training images look like regions in the test image and therefore the foetus is in the age range specified (see Supplementary Fig. 3 for an example).

In each stage, the participants examined 65 images chosen from the INTERBIO-21st dataset^66^ with an approximately uniform distribution of GA between 13–42 weeks (see Supplementary Fig. 5 and Supplementary Note 2 for further details). The study was performed online using the VGG Image Annotator (VIA) software^67^. The study received ethics approval from a subcommittee of the University of Oxford Central University Research Ethics Committee (Reference: R85756/RE001) and all participants gave written, informed consent.

It is important to discuss the expertise of the clinicians in the task of GA estimation from foetal ultrasound. Sonographers typically estimate GA using biometry, where structures, such as the circumference of the foetal head, are measured and compared to a standard growth chart^68,69^. This means the clinicians we recruit for the study are ideally suited for the task, in that they are the most qualified to estimate GA, but they are not trained to estimate GA from solely image characteristics, so cannot be considered experts at this particular task, because no experts exist.

### Model Development

We use a single model throughout the study, which outputs GA estimates and explanations. The estimates used in stage 2 are the same as those in stage 3, but in stage 3 the explanations are also displayed. For the model, we use an adaptation of an interpretable prototype-based deep learning model: prototypical part network (ProtoPNet)^45^. ProtoPNet classifies an image by calculating its similarity to a set of sub-parts of images from the training dataset and then weighting those similarities. This provides an explanation similar to how a clinician might make a prediction, e.g. “this foetus is 30 weeks of gestation, because it looks like a 30 week foetus I have seen before”. Below, we provide a summary of changes we made to ProtoPNet to make it more suitable for GA estimation. For details on model training and development see Supplementary Note 1.

ProtoPNet is designed for classification tasks, but GA estimation is a regression task. As such, we split the GA range from 13–42 weeks into 13 bins of approximately two weeks (the first and last bins were larger to account for less samples in this region). This means the model gives estimates in two-week intervals rather than a single number (e.g., 18–20 weeks).

ProtoPNet’s regularisation is not sufficient to arrive at simple explanations, as the number of prototypes (65) far exceed the number of explanations that can be reasonably presented to a clinician. We enforce a sparse model by pruning weights below some threshold, τ, and performing fine-tuning. By setting a τ of 0.25, we display a mean of 80% of the model’s reasoning with only four prototypes, compared to 42% for an unpruned model. For justification of this level of pruning see Supplementary Note 1.

The ProtoPNet model typically requires prototypes to be relevant to only a single class. Since GA estimation is a regression task converted into classification by binning, our classes have overlap in useful features. Hence, we remove the restriction that each prototype must be relevant to a single class, assuming prototypes are likely to be useful across a range of classes (Supplementary Fig. 4 provides some evidence for this hypothesis).

### Trust and reliance

To measure participant trust in the model during stages 2 and 3 we use a questionnaire based on work by Hoffman et al.^70^ to measure self-reported trust.

To measure reliance, we use two metrics evaluating participant agreement with model predictions. The first of these we simply name “agreement” and it is the proportion of estimates for which a participant’s estimate was within the GA range the model predicted. An increase in agreement indicates increased reliance. The second is an established measure of reliance: Weight of Advice (WoA)^59,71–75^. Many authors refer to WoA as a measure of trust^59,71–75^, but using our terminology, it measures reliance. First established to measure hindsight bias^76^, it measures the degree to which advice influences a participants estimate. Let $y$ be the ground truth GA, ${\hat{y}}_{{p}_{1}}$ be a participant’s estimate prior to observing information from the model, ${\hat{y}}_{{p}_{2}}$ be a participant’s estimate after observing information from the model, and ${\hat{y}}_{m}$ be the model’s estimate. In Eq. 1, we define WoA.

1 $${WoA}=\frac{{\hat{y}}_{{p}_{1}}-{\hat{y}}_{{p}_{2}}}{{\hat{y}}_{{p}_{1}}-{\hat{y}}_{m}}$$

Similar to Ahn et al.^75^, we do not include datapoints in the calculation of WoA where |${\hat{y}}_{{p}_{1}}-{\hat{y}}_{m}$| $< 1$. The justification for this is the 2-week intervals the model uses for its estimates. These intervals mean that if a participant’s estimate is within a week of the model’s estimate, then the participant and model agree. In general, a higher WoA indicates greater reliance. For a more detailed description of the meaning of different WoA values see Table 2.

Blind reliance on an inaccurate model can lead to negative outcomes. Instead, we want to achieve *appropriate reliance*, where participants rely on the model when it is correct but ignore it when incorrect^36–39^. Previous works define appropriate reliance (even if they use a different term like appropriate trust) using binary assignments of whether the model was correct or incorrect^38,39,77^. For a regression task, how close to the ground truth does a model estimate need to be for it to be correct? Rather than imposing an arbitrary correctness threshold (e.g., a fixed distance to ground truth), which may not generalise across tasks, we define correctness relationally—comparing the model to the participant’s own unaided estimate. If the model is closer to the ground truth than the participant, then the participant should rely on the model. If the model is less accurate, it is preferable to ignore it.

More concretely, we propose the following mathematical definitions of appropriate reliance, under-reliance, and over-reliance. The error for each estimate is the absolute difference with the ground truth:

2 $${\epsilon }_{{p}_{1}}=\left|{\hat{y}}_{{p}_{1}}-y\right|,{\epsilon }_{{p}_{2}}=\left|{\hat{y}}_{{p}_{2}}-y\right|,{\epsilon }_{m}=\left|{\hat{y}}_{m}-y\right|$$

Let ${{\rm{\delta }}}_{1}$ and ${{\rm{\delta }}}_{2}$ be the absolute difference between the participant’s estimate and the model estimate:

3 $${\delta }_{1}=\left|{\hat{y}}_{{p}_{1}}-{\hat{y}}_{m}\right|,\,\,{\delta }_{2}=\left|{\hat{y}}_{{p}_{2}}-{\hat{y}}_{m}\right|$$

Let ${\mathcal{R}}$ be a binary value indicating reliance:

4 $${\mathcal{R}}=\left\{\begin{array}{ll} 1 & {\text{if}}\, \delta_{2} < \delta_{1} \\ 0 & \text{otherwise} \end{array}\right.$$

That is, ${\mathcal{R}}=1$ indicates the participant moved closer to the model’s prediction, i.e., they relied on the model. We define ${{\mathcal{E}}}_{m}$ to indicate whether the model estimate was more accurate that the participant’s initial estimate:

5 $${{\mathcal{E}}}_{m}=\left\{\begin{array}{ll} 1 & {\text{if}}\, \epsilon_{m} < \epsilon_{p_1} \\ 0 & {\text{otherwise}} \end{array}\right.$$

We define reliance type, ${{\mathcal{R}}}_{t}$, by comparing behaviour (${\mathcal{R}}$) to whether reliance was warranted (${{\mathcal{E}}}_{m}$):

6 $${{\mathcal{R}}}_{t}= \left\{\begin{array}{lll} {\text{Appropriate Reliance}} & {\text{if}} \; {\mathcal{R}} = {\mathcal{E}}_m \\ {\text{Under-reliance}} & {\text{if}} \; {\mathcal{R}} = 0 \land {\mathcal{E}}_m = 1 \\ {\text{Over-reliance}} & {\text{if}} \; {\mathcal{R}} = 1 \land {\mathcal{E}}_m = 0 \end{array}\right.$$

It is worth restating that this definition does not assess whether the participant’s estimate improved, but rather whether their behaviour was justified given the model’s relative accuracy:

- Appropriate reliance: participant relied on the model when it was better, or did not when it was worse
- Under-reliance: participant did not rely on the model when it was better
- Over-reliance: participant relied on the model when it was worse

**Fig. 5:**
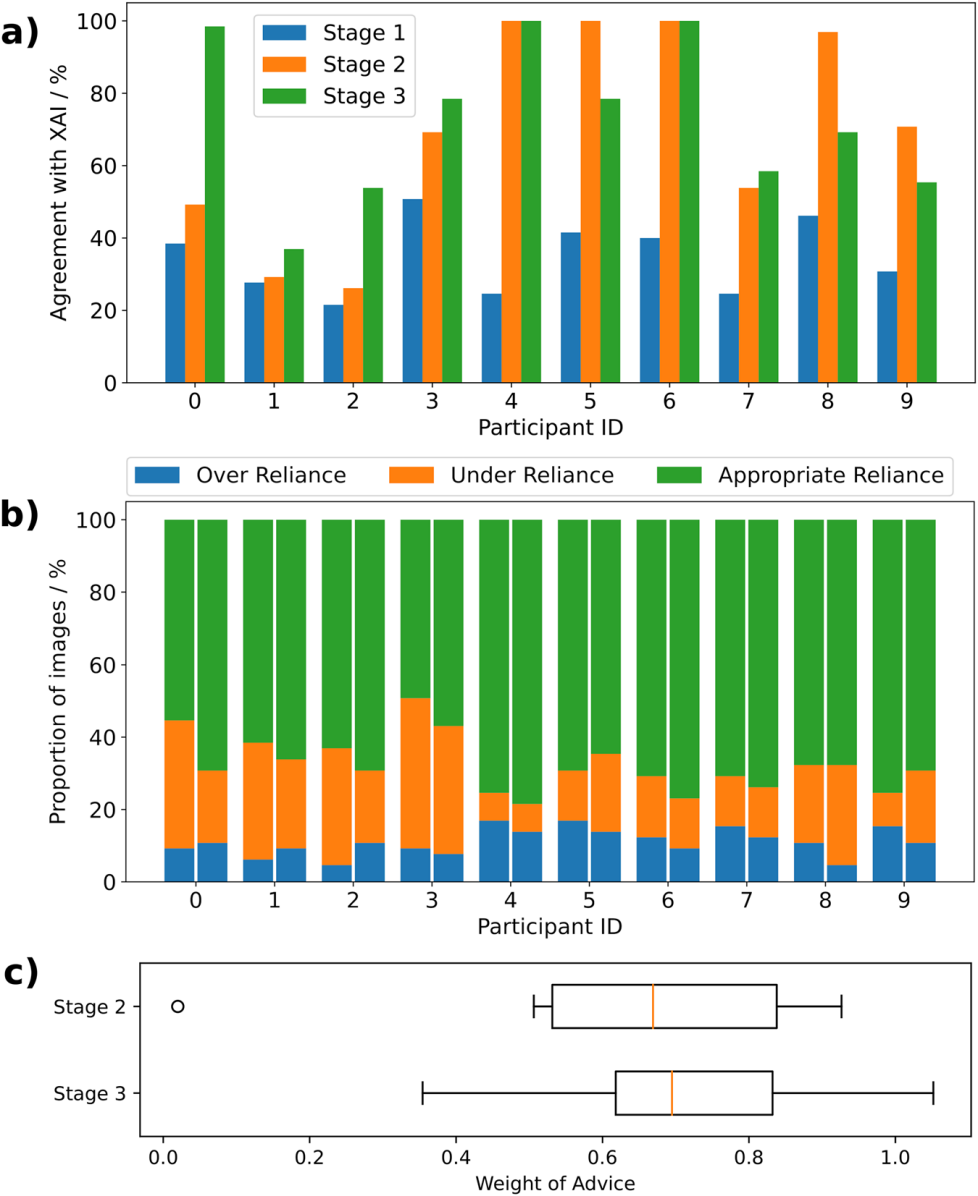
Participant reliance on the XAI model. **a** Participant agreement with XAI predictions for stages 1–3, i.e., the proportion of the time the participants’ predictions were within the model’s suggested range of GA. **b** The proportion of images for which participants showed over/under/appropriate reliance in the model for stages 2 and 3. **c** The mean Weight of Advice (measurement of reliance) of participants in Stage 2 and 3, where the box extends from the first quartile to the third quartile (Q3) and the orange line indicates the median value. The whiskers extend from the box to the farthest data point lying within 1.5x the inter-quartile range from the box, and outliers are plotted individually.

For understanding which type of reliance is most prevalent in a study, we follow Wang et al.^38^ by measuring appropriate reliance, over-reliance and under-reliance as a proportion of images/cases belonging to each reliance type (as in Fig. 5b).

While our definition of appropriate reliance is tailored to regression, the framework could naturally extend to other settings, such as classification tasks with probabilistic outputs or situations where confidence scores are available. In such cases, reliance could be defined using shifts in predicted probabilities or confidence movements toward the model.

## Supplementary information

Supplementary Information

## Data Availability

The consent forms for the study do not allow sharing of the study data, however study documents such as the study protocol, or participant questionnaires can be made available upon reasonable request to the corresponding author.

## Code availability

The underlying code for this study is available on GitHub and can be accessed via this link https://github.com/AngusNicolson/AGE-Study.
