# Supplementary material for: The human factor in explainable artificial intelligence: clinician variability in trust, reliance, and performance
Source: NPJ Digit Med. 2025 Nov 14;8:658. doi: 10.1038/s41746-025-02023-0 (PMC12618665; doi:10.1038/s41746-025-02023-0)
Supplement: Supplementary file 1 — Supplementary Information [file 41746_2025_2023_MOESM1_ESM.pdf]

Supplementary Information

The Human Factor in Explainable Artificial Intelligence: Clinician Variability in Trust, Reliance, and Performance

Angus Nicolson[1,2,†*], Elizabeth Bradburn[3,†], Yarin Gal[2],

Aris T. Papageorghiou[3,4,‡], J. Alison Noble[1,‡*]

*Corresponding author, † Joint first authors, ‡ Joint senior authors

1. Institute of Biomedical Engineering, Department of Engineering Science, University of Oxford, Oxford, UK

2. OATML, Department of Computer Science, University of Oxford, Oxford, UK

3. Nuffield Department of Women's and Reproductive Health, University of Oxford, Oxford, UK

4. Oxford Maternal & Perinatal Health Institute, Green Templeton College, University of Oxford, Oxford, UK

Corresponding authors:

Angus Nicolson, Email: angus.nicolson@eng.ox.ac.uk

J. Alison Noble, Email: alison.noble@eng.ox.ac.uk

Supplementary Note 1 – Model Training and Development

ProtoPNet

A ProtoPNet[45] model consists of a convolutional network, f , a prototype layer, g_p, and a fully-connected layer, h . In our experiments the convolutional network is a ResNet-18[78] pretrained on ImageNet followed by two 1×1 convolutional layers to reduce the number of output channels to 128.

The model makes a prediction by passing some input image, x , through the convolutional feature extractor to obtain a set of feature maps, $f(x)$, with shape $H \times W \times D$. The network learns m prototypes, $P = \{p\}_{j=1}^{m}$, which are vectors of shape $H_1 \times W_1 \times D$, where $H_1 < H$ and $W_1 < W$. In our experiments the feature maps are of shape $7 \times 7 \times 128$ and the prototypes $1 \times 1 \times 128$, so each prototype is a representation of some prototypical sub-patch of the image $\frac{1}{49}$th of its size. The prototype layer then calculates the L2 distance between each prototype and all 49 patches of the feature map. These distances are inverted to obtain a set of similarity scores and the maximum score for each prototype is then passed through the fully-connected layer. The maximal similarity scores can be seen as the likelihood that each prototype is present in the image and the weights in the fully-connected layer the importance of each prototype for each class. See Supplementary Figure 6 for a diagram of the architecture.

The model is globally interpretable because the prototypes and weights are fully accessible. Its local explanations consist of the prototypes most similar to a test image and their corresponding contributions to the model output.

The training protocol has three steps: (1) the prototypes and convolutional layers are jointly optimised using stochastic gradient decent (SGD); (2) the prototypes are pushed to the activations of the nearest (measured in feature space) image sub-patch of the train dataset - this provides the model its interpretability, as the prototypes can now be represented in image space by that sub-patch; (3) the rest of the network is frozen and the fully-connected layer is optimised using SGD. These three steps are repeated multiple times until the model converges.

The loss optimised during step (1) is composed of two parts: cross entropy and cluster loss. Cluster loss encourages the model to have at least one training sub-patch with activations close to each prototype. This minimises the changes made to the prototypes in step (2) of training. In step (3) the loss for h is cross entropy and an L1 loss to regularise the weights. For a detailed explanation of the losses, architecture and training protocol see the ProtoPNet paper[45].

In our work, compared to the ProtoPNet paper[45], the restriction that each prototype be relevant to a single class was removed. This is because the task of GA estimation is a regression task converted into a classification by binning, making the classes more similar to each other than in a standard classification task. Thus, it could be desirable for a prototype to be similar to multiple classes. We achieve this by removing the separation loss; the mask applied to the L1 regularisation and the cluster loss; and the class-dependent initialisation of the final layer. ProtoPNet[45] masked the L1 loss to reduce the level of negative reasoning present in the model,

the argument being it is easier to interpret the model if solely positive reasoning is used. Therefore, to increase the relative levels of positive reasoning in our model, we initialised the final layer to a uniform distribution between 0 and 1.

Pruning

We prune the final layer model weights by simply setting each weight below some threshold, τ , to zero. If all connections to a prototype are zero, the prototype is removed from the network. The fully-connected layer is then trained for 15 epochs, keeping any weights at zero fixed to allow the model to adapt to the changes, while keeping the same level of sparsity.

To measure the sparsity (simplicity) of the model, we define the number of relevant prototypes, r , in equation (2).

$$r = \frac{1}{K}\sum_{k=1}^{K}|P_k|. \qquad (2)$$

where $k \in \{1, \ldots, K\}$ is the class index. This is equivalent to the number of non-zero weights in the fully-connected layer divided by the number of classes. r gives an intuitive measure of the size of the model's global explanations as it is the average number of prototypes which affect each logit output. We also define r^+ and r^-, which are the subset of prototypes which have positive or negative weight connections, respectively. Similarly, we report the L1 loss on the subset of weights that are positive, $L1^+$, and negative, $L1^-$. These metrics allow us to track the relative level of positive/negative reasoning in the model.

Supplementary Figure 7 shows how the model performance and sparsity changes as τ increases. As the pruning threshold increases from 0 to 0.20, the MAE of the model does not change while the sparsity increases substantially, with L1 decreasing from 80.1 to 40.0 and r decreasing from 65.0 to 7.5. As τ increases beyond this, the MAE begins to increase, while the sparsity continues to increase. In order to retain good performance of the model while ensuring sparse explanations, we use a threshold of 0.25.

The explanations would be too complex if all prototypes are shown to the study participants each time. So, we must decide what subset to display. The obvious solution is to display the most salient prototypes, i.e. the prototypes which have the largest contribution to the predicted class logit output. But how many prototypes should we display? We display four prototypes as a reasonable balance between providing a faithful explanation of the model but not overloading the participants with information. Supplementary Figure 8 shows the mean proportion of the predicted class logit (explanation completeness) that is explained by displaying the top-N number of prototypes for three different levels of pruning: none, $\tau = 0.20$ and $\tau = 0.25$. As expected, we can see that as the number of prototypes increases, a larger proportion of the model output is explained and as the level of pruning increases, the increase in explanation completeness occurs faster. Our decision to use a model with a $\tau = 0.25$ is in part because by displaying four prototypes to the participants this model explains on average 78.5% of the model output compared to just 43.1% for the unpruned model.

Supplementary Figure 4 shows the mean contribution of each prototype to each class logit output for both the pruned ($\tau = 0.25$) and unpruned model. Each colour represents the contribution of each prototype. The fact that a single colour is often present for multiple adjacent classes indicates that our hypothesis that prototypes could be useful for a range of GA, as opposed to a single class, is correct. The figure reveals the simplicity of the pruned model compared to the unpruned model as each class often has only a handful of colours present, and therefore only a handful of prototypes which contribute to that classes output, as opposed to the unpruned model where each class depends on many prototypes. The lack of negative reasoning in the unpruned model is also apparent as only a single prototype appears below the x axis, meaning only that prototype has a negative contribution to model outputs.

Model Details

We used 5 prototypes per class, for a total of 65 initial prototypes. After pruning with a τ of 0.25, 34 prototypes remained and the L1 loss was reduced from 80.1 to 40.0. The model achieved a MAE of 6.84 days on a held-out INTERGROWTH-21st test set and 7.66 days on the INTERBIO-21st dataset. For the 65 images selected in the study, the model had a MAE of 9.42 days. In Supplementary B, we describe how these images were chosen. By chance, the images are substantially harder than the main dataset with a MAE of almost 2 days higher than the rest of INTERBIO-21st.

Supplementary Note 2 – Dataset Characteristics

Fetal head images from the INTERGROWTH-21st dataset[68,69] were used to train the XAI model. The dataset is from a healthy cohort of women from 8 different countries (Brazil, China, India, Italy, Kenya, Oman, UK, USA) who had known gestational ages via agreement between biometry measurements at first scan and last known menstrual period[79]. Each scan was done on the same model of scanner and using the same protocol. Multiple images per participant were obtained, with 4-5 visits at different gestational ages and a mean of 6 images per visit. Repeat images at a single time-point often differ only slightly in appearance.

The INTERBIO-21st dataset[66] is similar to the INTERGROWTH-21st dataset but with a different cohort of women and from a different set of countries (Brazil, Kenya, Pakistan, South Africa, Thailand, UK). The two cohorts have very different risk profiles due to their inclusion criteria. INTERGROTH-21st requires healthy women as participants whereas INTERBIO-21st includes some women in resource-poor settings at high risk for intrauterine growth restriction/small for gestational age and preterm delivery because of malnutrition and/or infection (HIV and malaria). This difference in cohorts is ideal for testing model performance in a different setting and proves a valuable validation set for the model.

We used only 65 images from INTERBIO-21st for the study. The 65 images were selected by manually examining 20 images from each age bin (a total of 260 images) and excluding images which did not show the complete head or were excessively zoomed out. We also excluded all images from Pakistan, since text containing information about the images obscured parts of the fetus. From the remaining images, we chose the 65 images which minimised the entropy of the

age distribution, i.e., the images which gave an age distribution closest to uniform across 13-42 weeks.

In Supplementary Figure 5 the gestational age distribution for our subset of the INTERGROWTH-21st dataset is shown, binned into the same classes as used for model development, along with the distribution for the INTERBIO-21st images used in the study. In both cases, the distribution is approximately uniform across 13 to 42 weeks with slightly less images at the extremes. Class imbalance is a known issue in training supervised deep learning models, but it is often an inherent feature of medical datasets. Through careful preprocessing, the effects of the imbalance can be reduced.[80] Hence, to reduce the imbalance in our dataset, the outermost classes were increased in size (13-16 and 38-42).

Supplementary Note 3 – Statistical Results

To explore *why* some participants improve in performance when they have access to model explanations in stage 3 and some do not, we evaluated the association between different factors and the change in MAE between stages 2 and 3. The factors tested include participant demographic information (age, years of experience in fetal ultrasound, frequency of obstetric ultrasound scans), pre-stage 1 question answers (related to trust, prior opinions on AI, usage of AI in current practice and familiarity with heatmaps), post-stage 2 question answers (related to trust in the model and the participant's usage of the model predictions), and post-stage 3 question answers (related to trust in the model, the participants usage of the explanations and their post-study opinions of AI). We used linear least squares regression and found that for most factors there was no significant association. For a full list of the factors and statistical results see Supplementary Table 1.

We additionally evaluated whether there was a change in any of these factors between the group of participants who self-reported that they found the explanations useful (n=4) and those who did not (n=6). Although there were some large effect sizes, we found very few statistically significant differences between the groups – likely due to the small number of participants in the study. For example, the Cohen's d for participant agreement to the statement "I would feel confident using a machine learning-based algorithm without any explanation" was 1.73, indicating that participants who felt like explanations are not necessary also found the explanations to be unhelpful, but the p value was 0.06 so we cannot rule out the null hypothesis of no difference in response between the two groups. In addition, participants who found the explanations helpful in stage 3 tended to think the explanations were less accurate in stage 2 (Cohen's d=1.68, unhelpful mean agreement = 4.17 ± 0.41, helpful mean agreement = 3.25 ± 0.96 to the statement "I felt the algorithm's estimates were accurate") but the difference was not significant (p=0.067 by independent T-test). We speculate that larger studies would find differences in some of these factors between the groups. For a full list of the factors and statistical results see Supplementary Table 2.

Supplementary Tables

Supplementary Table 1: Statistical results for linear least squares regression between the listed factors and the change in participant MAE between stages 2 and 3. Ordered by p value. In each case, the stage in which it was collected is listed. A negative r indicates that the error decreased as the factor increases (in the case of questions, this means as a participant agrees with the statement to a greater extent).

Factor	r	p
Post-Stage 3: I found the explanations helpful in making my estimates	-0.740	0.014
Post-Stage 3: I found the explanations helpful for all of the images	-0.740	0.014
Post-Stage 3: I found the explanations increased my level of trust in the model's estimate	-0.644	0.045
Post-Stage 3: The algorithm's estimate was helpful in my decision making	-0.622	0.055
Post-Stage 3: I found the explanations interesting	-0.588	0.074
Post-Stage 2: I felt the algorithm's estimates were accurate	0.540	0.107
Post-Stage 2: I trust machine learning-based algorithms	-0.523	0.121
Post-Stage 3: Providing the explanations would be useful for clinical decision making	-0.432	0.212
Post-Stage 2: I would be comfortable incorporating the algorithm into my clinical practice	0.425	0.221
Post-Stage 2: I distrust the algorithm	-0.402	0.249
Post-Stage 2: I distrust machine learning-based algorithms	-0.387	0.270
Pre-Stage 1: I understand the meaning of the term machine learning	-0.374	0.286
Pre-Stage 1: How many years have you been performing fetal ultrasound for?	-0.373	0.288
Pre-Stage 1: Strongly Disagree with: I am familiar with using image heatmaps for image analysis	0.367	0.297
Post-Stage 2: I am confident in the algorithm. I feel that it works well	0.348	0.325
Post-Stage 3: I would feel confident using a machine learning-based algorithm without any explanation	0.342	0.334
Post-Stage 2: I would be comfortable incorporating machine learning-based algorithms for gestational age estimation into my clinical practice	-0.339	0.338
Post-Stage 2: I would be comfortable incorporating machine learning-based algorithms into my clinical practice	-0.324	0.360
Post-Stage 3: I felt the algorithm's estimates were accurate	-0.313	0.378
Pre-Stage 1: I am familiar with using image heatmaps for image analysis	-0.306	0.390
Post-Stage 3: I distrust the algorithm	0.281	0.431
Post-Stage 2: The algorithm's estimate was helpful in my decision making	0.278	0.437
Pre-Stage 1: How often do you perform obstetric scans?	-0.272	0.447
Post-Stage 3: I followed the algorithm's recommendations every time	-0.266	0.457
Post-Stage 2: I trust the algorithm	0.258	0.471
Post-Stage 2: The algorithm is very reliable. I can count on it to be correct all the time	0.258	0.471
Post-Stage 2: I am wary of the algorithm	-0.240	0.504
Post-Stage 3: The outputs of the algorithm are very predictable	0.234	0.515

Pre-Stage 1: Age Category	-0.234	0.516
Post-Stage 3: I would be comfortable incorporating machine learning-based algorithms for gestational age estimation into my clinical practice	0.213	0.556
Post-Stage 3: I am wary of the algorithm	0.182	0.615
Post-Stage 2: I like using the system for gestational age estimation	0.180	0.620
Post-Stage 2: I feel safe that when I rely on the algorithm I will get the right answers	0.155	0.670
Pre-Stage 1: I am comfortable using new technology	0.150	0.679
Post-Stage 3: I would feel confident using a machine learning-based algorithm where no-one (clinicians or engineers) could understand how it reached its estimate as long as it had been through rigorous testing	0.144	0.692
Pre-Stage 1: I am currently using machine learning-based algorithms to aid my clinical practice	-0.140	0.700
Post-Stage 2: The outputs of the algorithm are very predictable	0.118	0.745
Post-Stage 2: I followed the algorithm's recommendations every time	0.112	0.758
Post-Stage 3: I like using the system for gestational age estimation	0.078	0.831
Post-Stage 3: I would be comfortable incorporating machine learning-based algorithms into my clinical practice	-0.067	0.853
Post-Stage 3: I would be comfortable incorporating the algorithm into my clinical practice	-0.065	0.859
Post-Stage 3: I trust the algorithm	-0.043	0.906
Post-Stage 3: I distrust machine learning-based algorithms	0.033	0.928
Post-Stage 3: I trust machine learning-based algorithms	-0.025	0.944
Post-Stage 3: I am confident in the algorithm. I feel that it works well	-0.018	0.960
Post-Stage 3: I feel safe that when I rely on the algorithm I will get the right answers	-0.012	0.975
Post-Stage 3: The algorithm is very reliable. I can count on it to be correct all the time	0.009	0.981

Supplementary Table 2: Statistical results for the difference in means for the listed factors (via an independent T-test) between the group of participants who self-reported finding the explanations useful and the participants who did not. Ordered by p value. In each case, the stage in which it was collected is listed. A large positive Cohen's d value indicates a large effect size and that the values were larger for the unhelpful group.

Factor	p	Cohen's d	Explanations unhelpful	Explanations helpful
Post-Stage 3: I found the explanations increased my level of trust in the model's estimate	0.006	-2.93	1.67 ± 0.82	3.75 ± 0.96
Post-Stage 3: I found the explanations interesting	0.009	-2.71	2.67 ± 0.82	4.25 ± 0.50
Post-Stage 3: I would feel confident using a machine learning-based algorithm without any explanation	0.060	1.73	3.33 ± 0.82	1.75 ± 1.50
Post-Stage 2: I felt the algorithm's estimates were accurate	0.067	1.68	4.17 ± 0.41	3.25 ± 0.96
Post-Stage 3: I would feel confident using a machine learning-based algorithm where no-one (clinicians or engineers) could understand how it reached its estimate as long as it had been through rigorous testing	0.148	1.26	3.33 ± 1.21	2.00 ± 1.41

Post-Stage 3: I followed the algorithm's recommendations every time	0.150	-1.26	2.33 ± 1.03	3.50 ± 1.29
Post-Stage 3: Providing the explanations would be useful for clinical decision making	0.183	-1.15	2.83 ± 0.75	3.75 ± 1.26
Post-Stage 2: I distrust the algorithm	0.200	-1.10	2.17 ± 0.98	3.00 ± 0.82
Post-Stage 3: I would be comfortable incorporating machine learning-based algorithms for gestational age estimation into my clinical practice	0.200	1.10	3.83 ± 0.98	3.00 ± 0.82
Post-Stage 3: I distrust the algorithm	0.242	1.00	3.33 ± 0.52	2.75 ± 0.96
Pre-Stage 1: How many years have you been performing fetal ultrasound for?	0.242	-1.00	1.67 ± 1.21	2.50 ± 0.58
Post-Stage 3: The algorithm's estimate was helpful in my decision making	0.242	-1.00	3.00 ± 1.10	3.75 ± 0.50
Post-Stage 3: I am wary of the algorithm	0.242	1.00	3.67 ± 0.52	3.25 ± 0.50
Post-Stage 2: The algorithm's estimate was helpful in my decision making	0.261	0.96	4.17 ± 0.75	3.50 ± 1.00
Post-Stage 3: I felt the algorithm's estimates were accurate	0.261	-0.96	2.83 ± 0.98	3.50 ± 0.58
Post-Stage 2: I distrust machine learning-based algorithms	0.311	-0.86	2.17 ± 0.75	2.75 ± 0.96
Pre-Stage 1: Strongly Disagree with: I am familiar with using image heatmaps for image analysis	0.312	0.85	0.83 ± 0.41	0.50 ± 0.58
Post-Stage 2: I would be comfortable incorporating the algorithm into my clinical practice	0.371	0.75	3.83 ± 1.33	3.00 ± 1.41
Post-Stage 3: The algorithm is very reliable. I can count on it to be correct all the time	0.393	-0.71	2.33 ± 0.82	2.75 ± 0.50
Post-Stage 3: I feel safe that when I rely on the algorithm I will get the right answers	0.393	-0.71	2.33 ± 0.82	2.75 ± 0.50
Post-Stage 3: I am confident in the algorithm. I feel that it works well	0.395	-0.71	2.67 ± 1.03	3.25 ± 0.96
Pre-Stage 1: Age Category	0.420	-0.67	1.83 ± 1.17	2.50 ± 1.29
Post-Stage 2: I trust the algorithm	0.447	0.63	3.50 ± 0.84	3.00 ± 1.15
Post-Stage 2: I would be comfortable incorporating machine learning-based algorithms into my clinical practice	0.462	-0.61	3.83 ± 0.75	4.25 ± 0.96
Post-Stage 2: I would be comfortable incorporating machine learning-based algorithms for gestational age estimation into my clinical practice	0.477	-0.59	4.17 ± 0.75	4.50 ± 0.58
Post-Stage 2: I trust machine learning-based algorithms	0.486	-0.58	3.50 ± 0.55	3.75 ± 0.50
Post-Stage 3: I would be comfortable incorporating the algorithm into my clinical practice	0.526	-0.52	2.83 ± 0.98	3.25 ± 0.96
Post-Stage 3: I trust the algorithm	0.526	-0.52	2.83 ± 0.98	3.25 ± 0.96
Post-Stage 3: I would be comfortable incorporating machine learning-based algorithms into my clinical practice	0.527	0.52	4.00 ± 0.00	3.75 ± 0.96
Post-Stage 2: I am wary of the algorithm	0.539	-0.51	3.33 ± 0.82	3.75 ± 1.26
Pre-Stage 1: I understand the meaning of the term machine learning	0.542	-0.50	3.67 ± 1.63	4.25 ± 0.96

Pre-Stage 1: How often do you perform obstetric scans?	0.610	0.42	0.50 ± 0.84	0.25 ± 0.50
Post-Stage 3: I trust machine learning-based algorithms	0.610	0.42	3.50 ± 0.55	3.25 ± 0.96
Post-Stage 3: I distrust machine learning-based algorithms	0.610	-0.42	2.50 ± 0.55	2.75 ± 0.96
Post-Stage 3: I like using the system for gestational age estimation	0.629	-0.40	3.00 ± 0.63	3.25 ± 0.96
Post-Stage 2: I am confident in the algorithm. I feel that it works well	0.645	0.38	3.33 ± 0.82	3.00 ± 1.41
Post-Stage 2: I like using the system for gestational age estimation	0.713	0.30	3.50 ± 0.55	3.25 ± 1.50
Post-Stage 3: The outputs of the algorithm are very predictable	0.721	-0.29	3.00 ± 1.10	3.25 ± 0.96
Pre-Stage 1: I am familiar with using image heatmaps for image analysis	0.735	-0.28	1.33 ± 0.82	1.50 ± 0.58
Pre-Stage 1: I am currently using machine learning-based algorithms to aid my clinical practice	0.760	-0.25	2.67 ± 1.03	3.00 ± 2.31
Post-Stage 2: I feel safe that when I rely on the algorithm I will get the right answers	0.767	0.24	3.00 ± 1.10	2.75 ± 1.50
Post-Stage 2: The outputs of the algorithm are very predictable	0.779	-0.23	3.33 ± 0.52	3.50 ± 1.29
Post-Stage 2: I followed the algorithm's recommendations every time	0.807	-0.20	3.00 ± 1.55	3.25 ± 1.50
Pre-Stage 1: I am comfortable using new technology	0.844	-0.16	3.83 ± 1.17	4.00 ± 1.41
Post-Stage 2: The algorithm is very reliable. I can count on it to be correct all the time	0.919	-0.08	2.67 ± 1.03	2.75 ± 1.50

Supplementary Figures

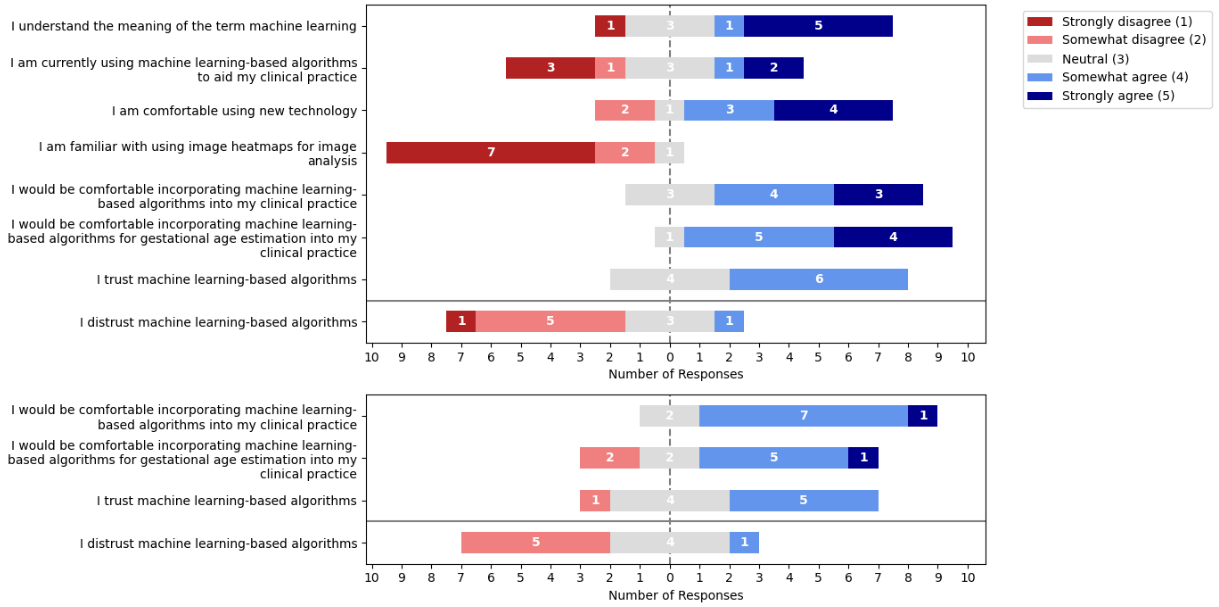

Supplementary Figure 1: Participant opinions on machine learning/AI before and after the study. Responses to “On a scale of 1-5, how much do you agree with the following statements?” at the beginning (top) and end (bottom) of the study on a Likert scale.

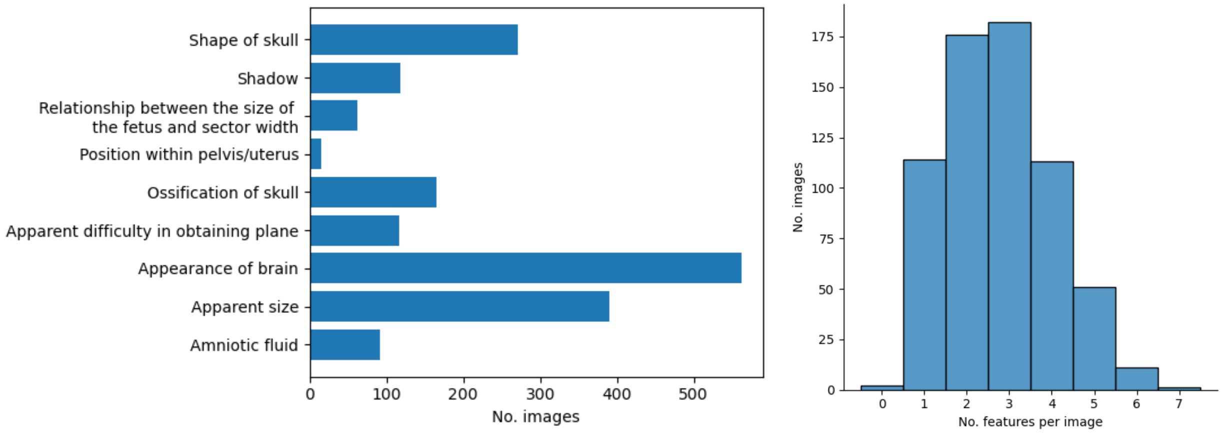

Supplementary Figure 2: Participants used a variety of image features to estimate GA. Left: The number of images for which participants found each feature to be useful for GA estimation in Stage 1 (left). Right: The distribution of the number of features participants selected per image.

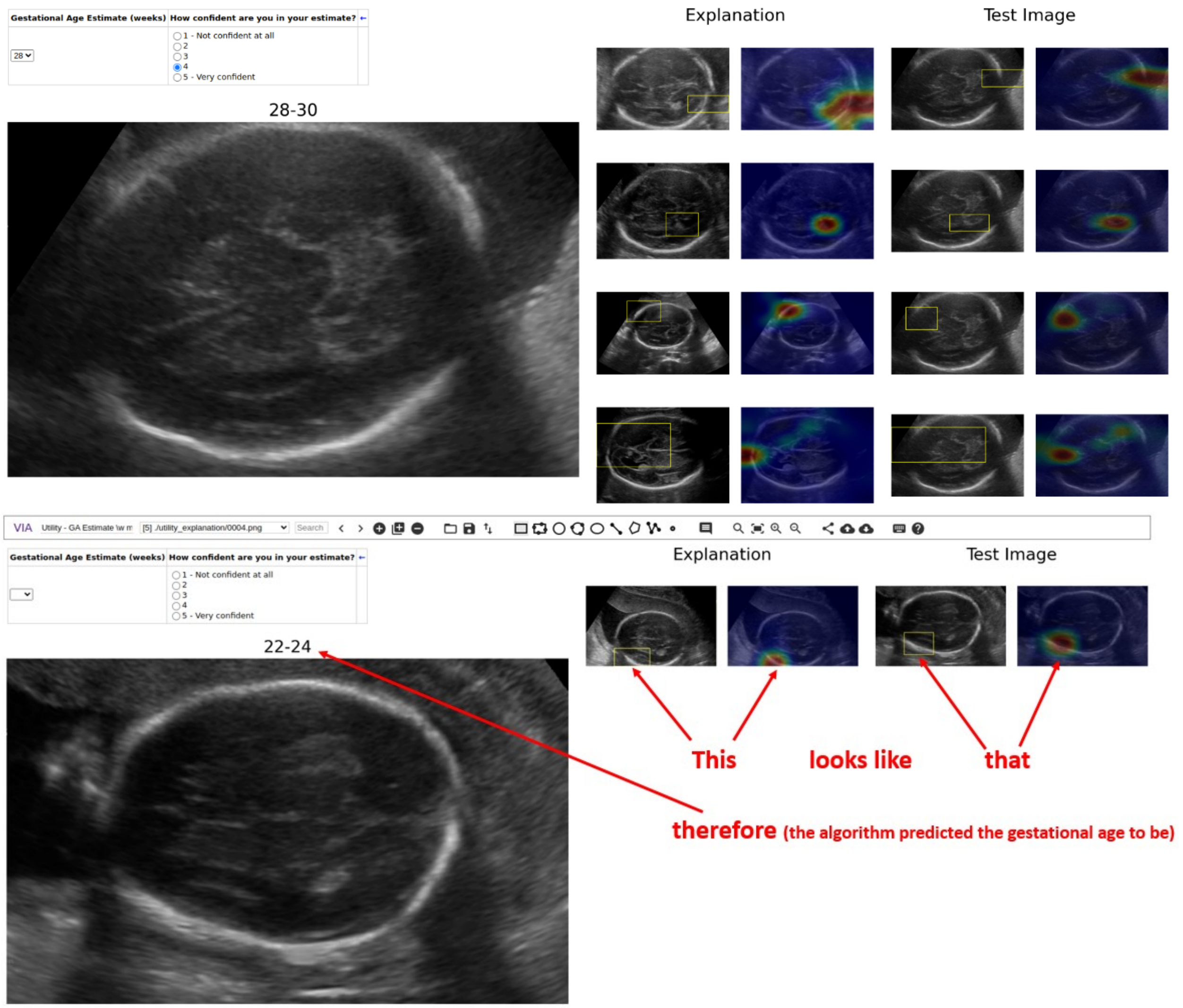

Supplementary Figure 3: Screenshots of the explanations used in the study. Top: Screenshot of the VIA software in stage 3 displaying the test image (left), model predictions (above the test image), questions for the participant (top left) and model explanations (right). Bottom: A second screenshot but with the method of interpreting the explanations overlaid.

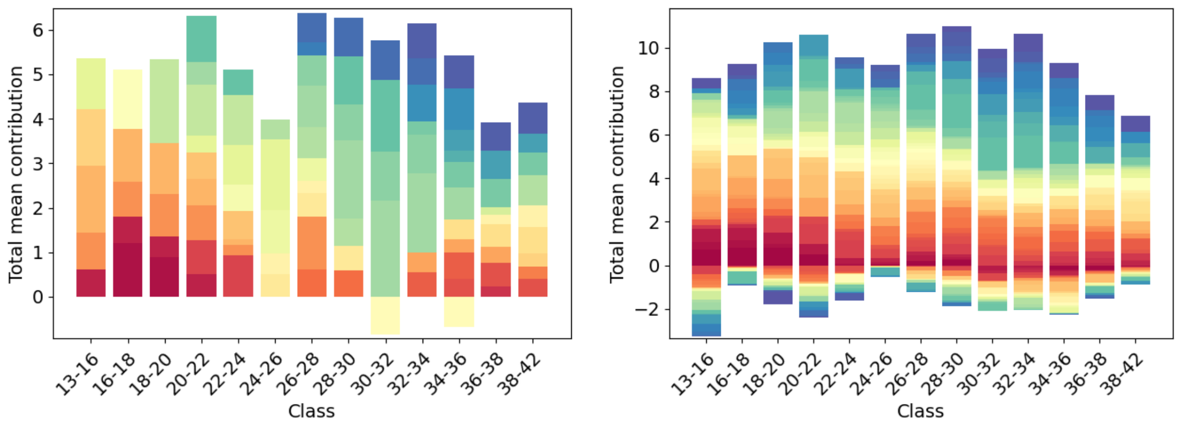

Supplementary Figure 4: Prototypes are used across similar ages. The mean contribution of each prototype to the logit output of each class for the pruned model used in the AGE study (left) and original unpruned model (right). Each colour represents a unique prototype's contribution, with negative contributions starting from from zero downwards and positive from zero upwards. The same colour used across a range of classes shows that prototypes tend to be used across a range of GA.

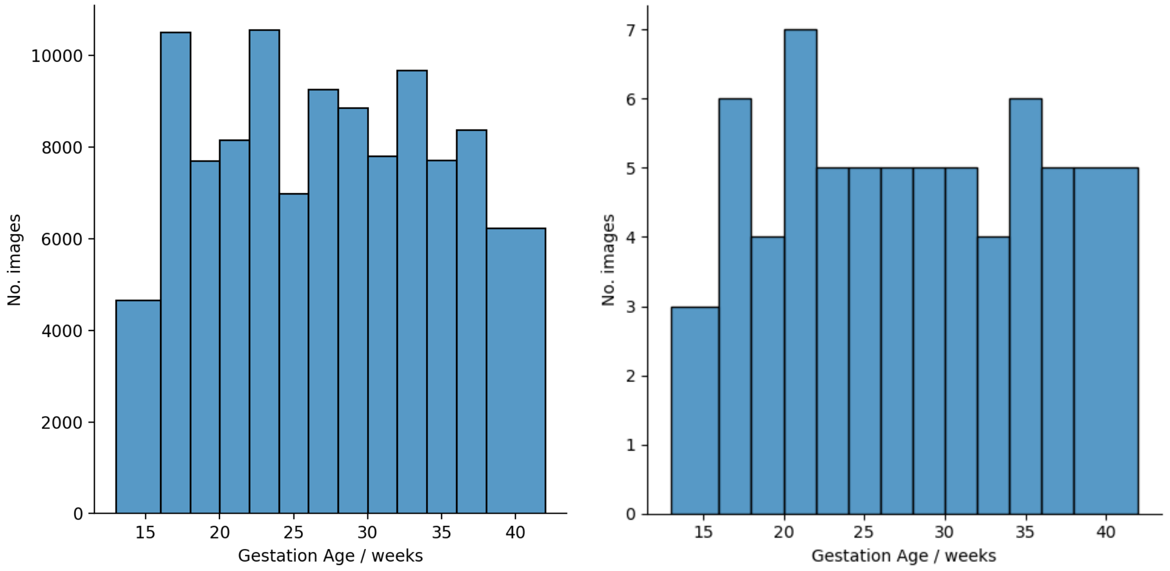

Supplementary Figure 5: Gestational Age distributions for the study datasets. GA distribution for INTERGROWTH-21st (left) and for the 65 images used in the study from INTERBIO-21st (right) binned by the classes used in the XAI model.

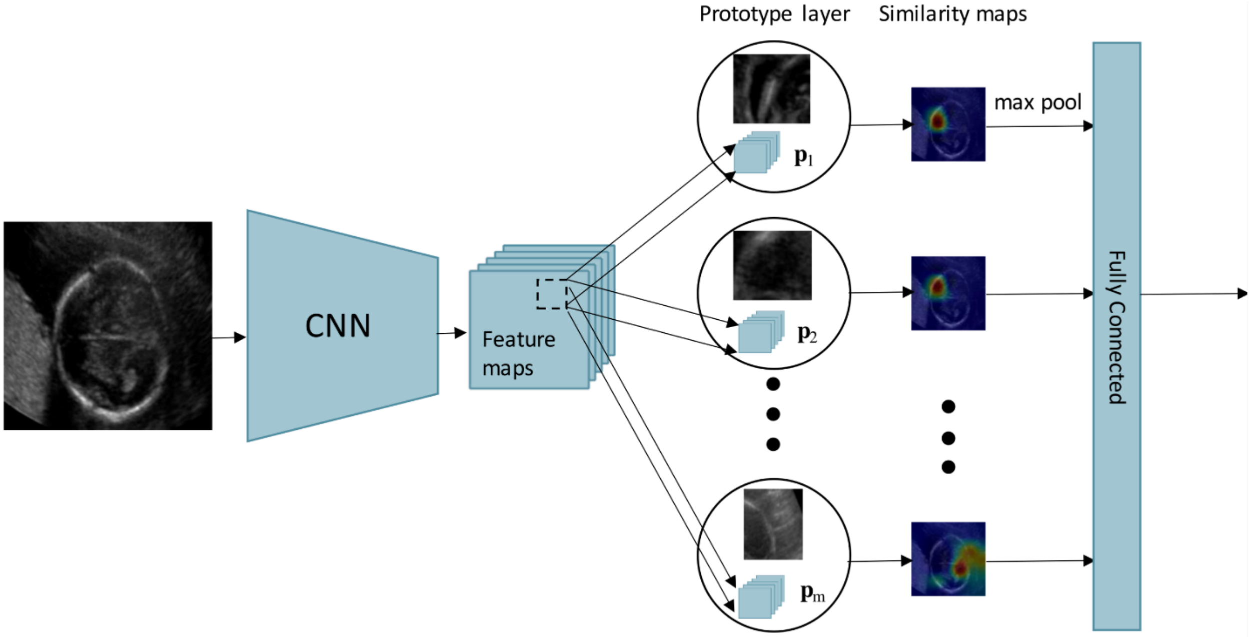

Supplementary Figure 6: Prototypical part network (ProtoPNet) architecture. A test image is passed through the convolutional neural network (CNN) backbone to obtain a set of feature maps. These feature maps are compared to the features of training images the model has seen previously, i.e. the prototypes, and similarity maps obtained. After a max pooling operation, the values are passed to a fully connected layer to classify the image. This means the predictions are made solely based on the similarities between the test image and the prototypes, making an interpretable-by-design model.

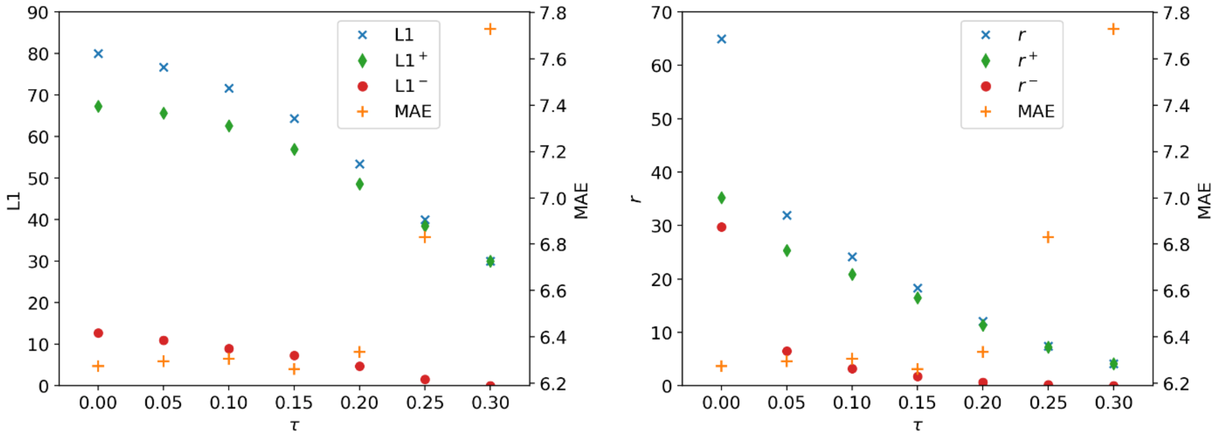

Supplementary Figure 7: Model results for INTERGROWTH-21st as the pruning threshold, τ , is increased. Fully connected L1 loss (left) and mean number of relevant prototypes, r , (right) for all (blue cross), positive (green diamond) and negative (red circle) weights against pruning weight threshold, τ . The model MAEs (orange plus) are shown on the right axis of each plot

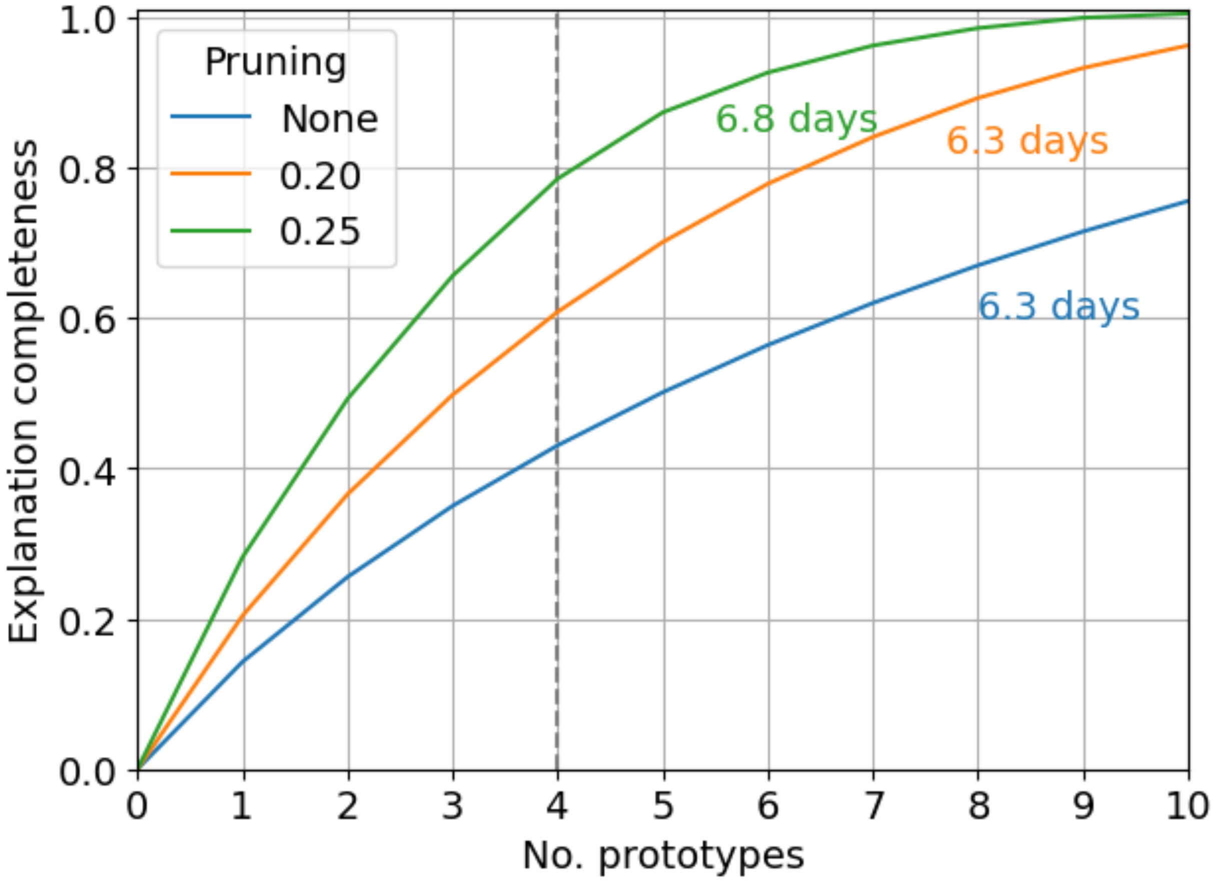

Supplementary Figure 8: Explanation completeness increases with more pruning. The mean proportion of a model's reasoning explained (explanation completeness) by the number of prototypes that are shown for a model with no (blue), moderate (orange) and high (green) pruning. The MAE for INTERGROWTH-21st for each model is shown next to each curve. As the models are pruned more, a greater proportion of the model is explained with fewer prototypes, but the model MAE increases.

Supplementary References

[78] He, K., Zhang, X., Ren, S. & Sun, J. Deep residual learning for image recognition. *CVPR* 770-778 10.1109/CVPR.2016.90 (2016).

[79] Villar, J. et al. The objectives, design and implementation of the INTERGROWTH-21st Project. *BJOG: An Intern. J. of Obstetrics Gynaecology*. **120,** Suppl. 2, 9-26 (2013).

[80] Hassler, A., Menasalvas, E., García-García, F. et al. Importance of medical data preprocessing in predictive modeling and risk factor discovery for the frailty syndrome. *BMC Med Inform Decis Mak*. **19,** 33 (2019)
